# Identification of a Single Nucleotide Polymorphism of Vitamin D Receptor (VDR) and Vitamin D Binding Protein (VDBP) Gene and Its Dysregulated Pathway Through VDR-VDBP Interaction Network Analysis in Vitamin D-Deficient Infertile Females

**DOI:** 10.7759/cureus.55602

**Published:** 2024-03-05

**Authors:** Zil E Rubab, Sumaira Naz, Mussarat Ashraf, Saba Shahid, Rehana Rehman

**Affiliations:** 1 Department of Biochemistry, Ziauddin University, Karachi, PAK; 2 Department of Obstetrics and Gynaecology, The Aga Khan University, Karachi, PAK; 3 Department of Biological and Biomedical Sciences, The Aga Khan University, Karachi, PAK

**Keywords:** smad3, ncori, female infertility, vdbp, vdr, plasma membrane estrogen receptor signalling pathway

## Abstract

Introduction: The prevalence of female infertility in Pakistan is currently estimated at 22%, and emerging research suggests that vitamin D (VD) deficiency (VDD) may play a significant role in influencing female fertility. The focus of this study was to investigate the single nucleotide polymorphism (SNP) patterns within the VD binding protein (VDBP). The study aimed to explore dysregulated pathways and gene enrichment through an interaction network analysis, specifically focusing on the interplay between the VD receptor (VDR) and VDBP in females experiencing unexplained infertility (UI) coupled with VDD.

Methods: A cross-sectional study was conducted on VD-deficient, fertile, and UI female subjects. VDBP and VDR were assessed by enzyme-linked immunoassay and genotyping performed. FunRich (version 3.1.3; http://funrich.org/index.html) was employed for analysis of the identified proteins: VDR and VDBP and with their mapped gene datasets, gene enrichment, and protein-protein interaction (PPI) network.

Results: The mean VD and VDR values of infertile females were significantly lower than those of fertile females. VDBP in infertile females (median (IQR)): 296.05 (232.58-420.23)) was lower than that of fertile females (469.9 (269.57-875.55), (p=0.01)). On sequence analysis, a mutation rs 4588 SNP (Thr 436 Lys) was found in exon 11 of the VDBP gene of UI females, but no mutation in exons 8 and 9 of the VDR gene, with some insignificant intronic variants, was observed. The proteins such as plasma membrane estrogen receptor signaling pathway (p < 0.001), VDR, SMAD3, NCOR1, CREBBP, NCOA1, STAT1, GRB2, PPP2CA, TP53, and NCOA2 were enriched after biological pathway grouping when VDR was made the focused gene and directly interacting with VDBP.

Conclusion: The females with UI exhibited significantly low VD, VDBP, and VDR. The plasma membrane estrogen receptor signaling pathway was enriched in VDD infertile females.

## Introduction

Vitamin D (VD) is involved in calcium-phosphate homeostasis and maintenance of bone mineral density. The active form for VD, namely, 1, 25-dihydroxyvitamin D3 (1, 25-(OH)2D3), metabolized by 1-alphahydroxylase from cholecalciferol (25-OHD), exerts these effects through the VD receptor (VDR). This receptor is present in the intestines, bones, parathyroid glands, ovaries, and testes [1]. Furthermore, it may be found along the walls of the central organs of reproduction such as the pituitary and hypothalamus and peripheral organs such as the oviduct, uterus, and placenta [2]. VD binds to VDR a transcription factor situated in the nuclei of target cells that facilitates the genomic action of the active form of VD (1,25(OH)2D3). This transcription factor is circulated to different tissues, functions as an ovarian reserve marker, and stimulates the production of hormones from the ovaries [3].

The probable role of VD in the impairment of reproductive physiology and the relationship of VD deficiency (VDD), with VDR polymorphism and infertility in female subjects, has been explored [4,5].

The VDR gene is positioned on chromosome 12 that extents around 75 kb of genomic DNA and comprises 11 exons [6]. An ongoing argument on global VDD and the presence of VDR in reproductive tissues with an increased prevalence of infertility has encouraged us to conduct the study.

Approximately one in eight women of reproductive age pursue advice for infertility issues, and 85% of these patients have an underlying cause. Among females, the common causes of infertility are anovulation, tubal disorders, and endometriosis. However, in 15% of infertile women, no definite cause is identified, which leads to the diagnosis of unexplained infertility (UI) [7]. UI is defined as the inability to conceive despite 12 months of unprotected intercourse, in the absence of known causes of infertility, including anovulation, tubal pathology, endometriosis, or semen abnormalities [8]. Many women diagnosed with UI may conceive spontaneously over a period of time at the rate of 2%-4% per menstrual cycle, whereas others need treatment with ovarian stimulation and intrauterine insemination; if these approaches are not successful, in vitro fertilization (IVF) is considered [7]. As UI is a diagnosis of exclusion, therefore, it is difficult to have any definite explanation of affected fertility potential in these patients.

As far as the association between VD and infertility is concerned, the association of polycystic ovarian syndrome and endometriosis with VDD and infertility has been established [9]. VD has been linked with successful outcomes in IVF outcomes as well [10]. Similarly, low levels of VD have been correlated with low pregnancy rates due to its harmful effects on conception and endometrial receptivity in women undergoing IVF with single embryo transfer. Therefore, VDD may explain some cases of UI or can be a contributing element to other factors that alter fertility potential. However, no conclusive data are available to examine the relationship between VD and UI or low ovarian reserve.

VDR exhibits widespread distribution across nearly all human tissues. Moreover, VD plays a regulatory role in the human genome, underscoring its potential influence on various systems, including reproductive processes [11]. Due to the VDR presence in ovaries and endometrium, the impact on UI could be at multiple levels. In ovaries, VD-mediated calcitriol has effects on ovarian steroidogenesis that stimulate hormone synthesis, including progesterone, estradiol, and estrone [12]. Furthermore, in vitro studies have shown an association of VD with ovarian reserve markers such as anti-Mullerian hormone (AMH) [13] and demonstrated the presence of functional VD response element (VDRE) on human AMH promoters [14]. One study indicated a significant reduction in AMHR-II and FSH receptor mRNA with human granulosa cells by VD3 [15]. As AMH has its inhibitory role on folliculo-genesis, this can be anticipated that VD treatment can have a beneficial role on folliculo-genesis by alleviating the inhibitory influence of AMH on the process.

We wanted to identify the single nucleotide polymorphism (SNP) of VD binding protein (VDBP), the dysregulated pathways, and gene enrichment centered on interaction network analysis of VDR-VDBP in unexplained infertile females with VDD.

The preprint version of the manuscript was published at https://doi.org/10.21203/rs.3.rs-1565925/v1.

## Materials and methods

This study was a cross-sectional study, and it was conducted from June 2019 to July 2020 after approval from the Institutional Ethical Review Committee (ERC) of Aga Khan University (AKU-ERC 2019-0314-5627) in association with the Australian Concept Infertility Medical Centre (ACIMC).

Inclusion criteria: In this study, we included fertile females between the age range of 18-45 years from all ethnic backgrounds and having a child with ages of less than two years. Recruitment of infertile females was based on the criteria of UI in the age range of 18-45 years and from all ethnic groups.

Exclusion criteria: Infertile females due to male factor causes or due to tubal blockade were excluded. Furthermore, female subjects with a previous history of artificial reproductive techniques (ART) in preceding pregnancies, recurring miscarriages, thyroid abnormalities, uterine tumors, hypertension, and diabetes were excluded. Infertile females with serious general health problems, using contraceptive pills orally and any hormonal treatments, or using any contraceptive procedures were also excluded. We also excluded women (fertile and infertile) who were on VD therapy, calcium supplementation (for the last six months), or exposed to tobacco, gonadotropins, or prior chemotherapy.

The history of the patient was acquired, and BMI was calculated by using South Asian criteria. 

Blood collection: For the recruitment of subjects, approximately 5 mL of venous blood sample was collected from each subject by a pain-free procedure. Serum was extracted by centrifugation of blood samples and quickly stored at -80 °C until estimation of biochemical parameters.

Biochemical analysis: VD levels in serum were measured by a human, 1,25-dihydroxy VD enzyme-linked immunosorbent assay (ELISA) kit (Cat No. 95503), with an intra- and inter-assay coefficient of variation (CV) of 2.7% and 4.3%, respectively. The lowermost limit of detection of VD was 2.8 ng/mL. After the detection of VD levels, 80 VDD females (40 in each fertile and infertile group) were recruited for the study. VDR and VDBP levels were analyzed in the comparative groups. VDR levels in serum were measured by a commercially available ELISA kit (Cat. No: SEA475Hu; Cloud-Clone Corp, Houston, TX) with a detection range of the kit of 0.625-40 ng/mL. The analytical sensitivity was less than 0.225 ng/mL, and intra- and inter-assay CVs were found to be <10% and 12%, respectively. VDBP levels in serum were observed by using a commercially available human VDBP ELISA kit (Cat. No: 96577; Glory Science Co. Ltd., Taichung, Taiwan) using a detection range of the kit of 8-480 ug/mL.

Genotyping

Genotyping of VDR was performed using the SNP genotyping assay, primers (Table 1), and direct DNA sequencing methods. These primers were designed by the Primer3 online tool (https://primer3.ut.ee/).

**Table 1:** Primer Sequence of VDR and VDBP VDR - Vitamin D receptor; VDBP - Vitamin D binding protein

S. No.	Primers	Primer Sequence
1	Exon8 F – VDR	GGTGTATACCTGTCAAAGCACTA
2	Exon8 R – VDR	CCCTGTTGGTGCCTAACTC
3	Exon9 F – VDR	GGGAGTTAGGCACCAACAG
4	Exon9 R – VDR	CCCTCAGCAGGTCTTTGTC
5	Exon 11-F – VDBP	TAATGAGCAAATGAAAGAAG
6	Exon 11-R – VDBP	TGAGTAGATTGGAGTGCATAC

Polymerase chain reaction (PCR) for exons 8 and 9 of the VDR gene was performed using a 2× PCR Master Mix (Cat. No. G013; Applied Biological Materials Inc., Canada) as per the manufacturer’s instructions. PCR conditions were initial denaturation at 95 °C for 5 min by one cycle and then further 35 cycles at 95 °C for 30 s, 58 °C for 45 s, and 72 °C for 45 s, which is then followed by a final extension at 72 °C of 10 min. PCR was performed for VDBP using the GoTaq hot start master mix (Cat. No. M5122; Promega Corporation, Madison, WI) as per the instructions provided. PCR conditions were initial denaturation at 95 °C for five min by one cycle and then further at 95 °C for 30 s, 60°C for one min, 72 °C for 45 s by 40 cycles, and followed by a final extension at 72 °C of 10 min. The amplified products were run on gel electrophoresis using 2% agarose gel. Purification of the PCR products was performed by using PCR clean-up of DNA sequencing (Cat. No. BT5100; Bio Basic Inc., Canada) using the instructions in the protocol.

Sanger sequencing is a classical method for sequencing. This method was utilized to sequence the VDR and VDBP genes in samples, and PCR products were sent to the sequencing company Operon Technologies Inc. (Alameda, Canada). Previously published VDR and VDBP gene sequence data were directly used to compare the resultant sequences using the National Centre for Biotechnology Information (NCBI) database MEGABLAST search engine. Sequence files were viewed by importing them into Chromas Lite and then analyzed by assembling them into Molecular Evolutionary Genetic Analysis (MEGA), version 6.0.

Statistical analysis was accomplished by using Statistical Product and Service Solutions (SPSS, version 20; IBM SPSS Statistics for Windows, Armonk, NY) software by performing descriptive statistics and the Mann-Whitney U test. Statistical significance was considered at a p-value of < 0.05. Sequences were analyzed by using MEGA software (version 6.0).

VDPB and VDR gene interaction pathways were studied in this in silico analysis.

Bioinformatic analysis

The VDPB and VDR protein interaction network, mapping of the gene datasets and interaction pathways, was obtained by FunRich (version 3.1.3; http://www.funrich.org), which is a functional enrichment analysis tool.

Enrichment analysis

Molecular functions, biological pathways, gene ontology (GO) terms, and sites of expression terms were retrieved by performing enrichment analysis. The depleted and enriched proteins were identified by the fold change for biological pathways, protein domains, and sites of expression.

Interaction network analysis

Biological pathway enrichment of defined nodes was used for visualizing and analyzing the protein-protein interaction (PPI) network. Only human-exclusive datasets were presented in which gene/protein annotations were collected from publicly available protein-centric and gene databases. The human-specific FunRich database was selected as the background database for complete analysis. The list of genes augmented in specific pathways was highlighted within the interaction network, and distinctive sub-networks were created for complete analysis. Specific sub-network was analyzed by tallying direct neighbors (interacting partners) for mentioned nodes in the sub-network and visualization. Specific nodes were focused, and the interacting partners of the focused nodes were mapped.

GO functional categories, normal and overrepresented and identified pathway associations, and significant interactions with datasets were analyzed by using the Benjamini-Hochberg (BH) and Bonferroni tests. The p-value correction was done with the BH and Bonferroni tests and hypergeometric test, and a p-value <0.05 was taken as the statistical cutoff and maintained as default after Bonferroni correction.

## Results

This cross-sectional study describes the SNP of VDBP, the dysregulated pathways, and gene enrichment centred on interaction network analysis of VDR-VDBP in UI females with VDD.

The demographic and biochemical characteristics of fertile and infertile female subjects (80, fertile=40 and infertile=40 females) are presented in Table 2. The mean age of the female subjects was comparable between fertile and infertile groups. The BMI (mean ± SD) was greater in infertile females (27.4 ± 3.6) as compared to fertile females (23.5 ± 1.7; p<0.001). The mean vitamin D values of infertile females (7.45 ± 2.1) were significantly lower than fertile females (15.82 ± 5.2; p=0.006). The mean VDR values of infertile females (27.32 ± 7.45) were also significantly lower than fertile females (41.25 ± 8.1; p<0.001). Levels of VDBP in infertile females (median (IQR), 296.05 (232.58-420.23)) were lower as compared to fertile females (median (IQR), 469.9 (269.57-875.55), (p=0.01)).

**Table 2:** Comparison of study variables in fertile and infertile females *VDR (Vitamin D receptor) - Values are expressed as mean ± SD, ** VDBP (vitamin D-binding protein) - Median (IQR). The Mann-Whitney U test was applied to find p-values, and p values <0.05 were considered statistically significant.

	Fertile (n=40)	Infertile (n=40)	P-value
Age (years)*	29.4 ± 6.2	31 ± 5.7	0.416
BMI (kg/m^2^)*	23.5 ± 1.7	27.4 ± 3.6	<0.001
Vitamin D (ng/mL)*	15.82 ± 5.2	7.45 ± 2.1	0.006
VDR (ng/mL)*	41.25 ± 8.1	27.32 ± 7.45	<0.001
VDBP (ng/mL)**	469.9 (269.57-875.55)	296.05 (232.58-420.23)	0.01
Estradiol (pg/mL)*	270.36 ± 275.72	277.76 ± 264.65	0.878

**Figure 1:**
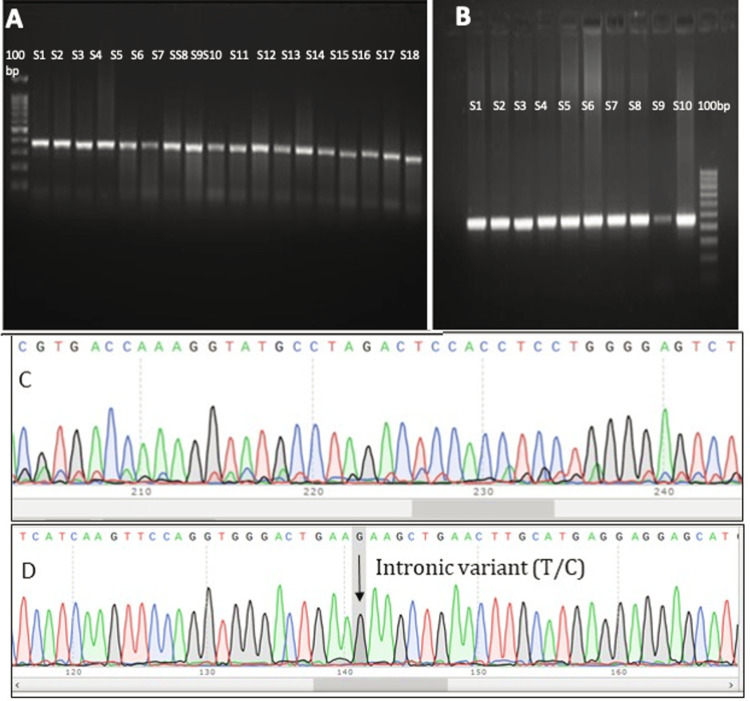
Gel electrophoresis of VDR and VDBP genes and sequencing chromatogram Figures 1A-1B: PCR amplification shown as bands on 2% agarose gel of VDR (355 bp) and VDBP (462 bp) of fertile and infertile subjects numbered S1-S18 and S1-S10, respectively. Figures 1C-1D: Sanger sequencing chromatograms of exons 8 and 9 of the VDR gene in infertile females, respectively, with highlighted intronic variant (T/C) in exon 9.

Figures 1A-1B represent gel electrophoresis images of amplified PCR products for the VDR gene (band size=~355 bp) and for the VDBP gene (band size=~462 bp) in infertile and fertile females. On sequence analysis, a mutation rs 4588 SNP (Thr 436 Lys) was found in exon 11 of the VDBP gene of infertile females, and a C/T mutation was found at position 4,7846,396 in exon 9 of the VDR gene of infertile females. Figure 1C represents the sequencing chromatogram of exon 9 of the VDR gene in infertile samples. Figure 1 D shows Sanger sequencing chromatograms of exons 8 and exon 9 of the VDR gene in infertile females, respectively, with highlighted intronic variant (T/C) in exon 9.

Interacting proteins for VDBP and VDR genes

**Figure 2:**
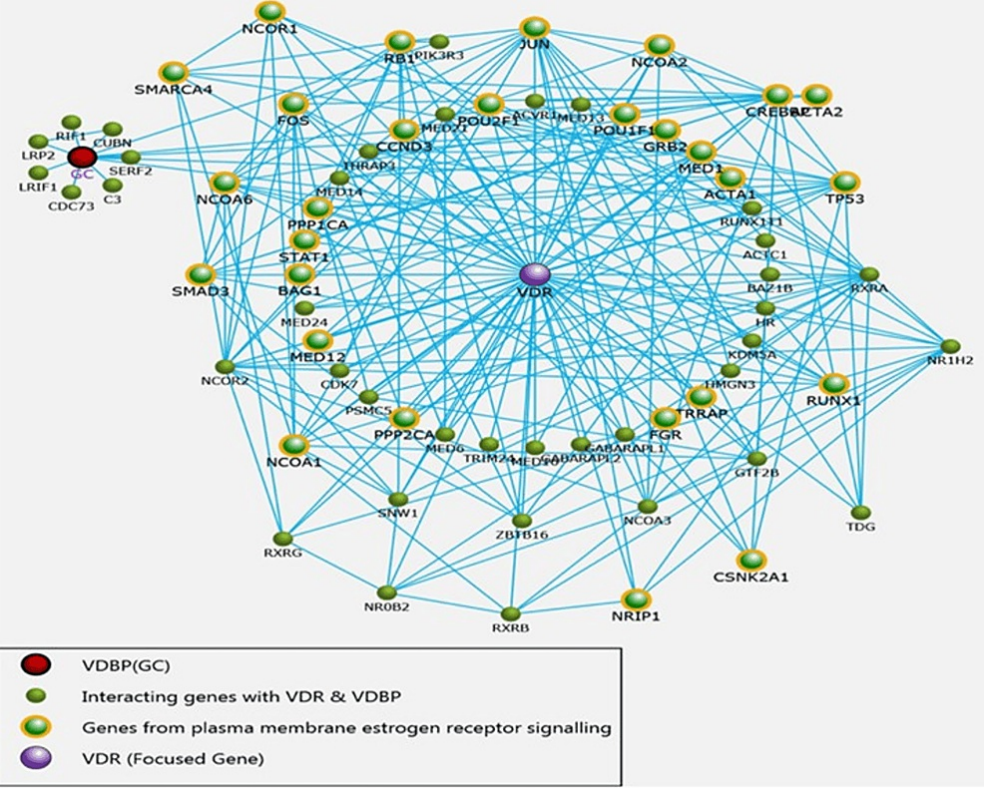
Protein-protein interaction (PPI) of VDR-VDBP (vitamin D receptor-vitamin D-binding protein) VDBP (GC) - Vitamin D-binding protein (group-specific component); VDR - Vitamin D receptor

FunRich (version 3.1.3) was employed for the analysis of the identified proteins: VDR and VDBP along with their mapped gene datasets, enrichment, and protein-protein interaction (PPI) network. There were 66 proteins identified interacting directly with VDR and VDBP (GC) (Figure 2).

PPI analysis of VDBP and VDR genes

The FunRich database was used to evaluate the PPI network and envisage the VDR-VDBP (GC) interaction. The interaction network was integrated into the pathway enrichment of the identified protein. The differentially controlled interacting proteins of potential retrieved from the interaction of VDR and VDBP (GC) were recognized in this network (Figure 2). Sixty-six genes were established interacting with VDR and GC, all interacting conjointly (Figure 2). The proteins described in the main group mapped along with VDR and VDBP were CREB-binding protein, nuclear receptor corepressor 1; mothers against decapentaplegic homolog 3; receptor-regulated SMAD (R-SMAD); nuclear receptor coactivator 3; and nuclear receptor coactivator 1.

The important and associated pathways with their interacting proteins were epidermal growth factor receptor (EGFR)-dependent endothelin signalling events, platelet-derived growth factor receptor (PDGFR)-beta signaling pathway, tumor necrosis factor-related apoptosis-inducing ligand (TRAIL) signaling pathway, plasma membrane estrogen receptor signaling, validated nuclear estrogen receptor beta network, retinoic acid receptors-mediated signaling, androgen-mediated signaling, regulation of androgen receptor activity, glucocorticoid receptor signaling, mTOR signaling pathway, TGF-beta receptor signaling, and regulation of cytoplasmic and nuclear SMAD2/3 signaling.

**Figure 3:**
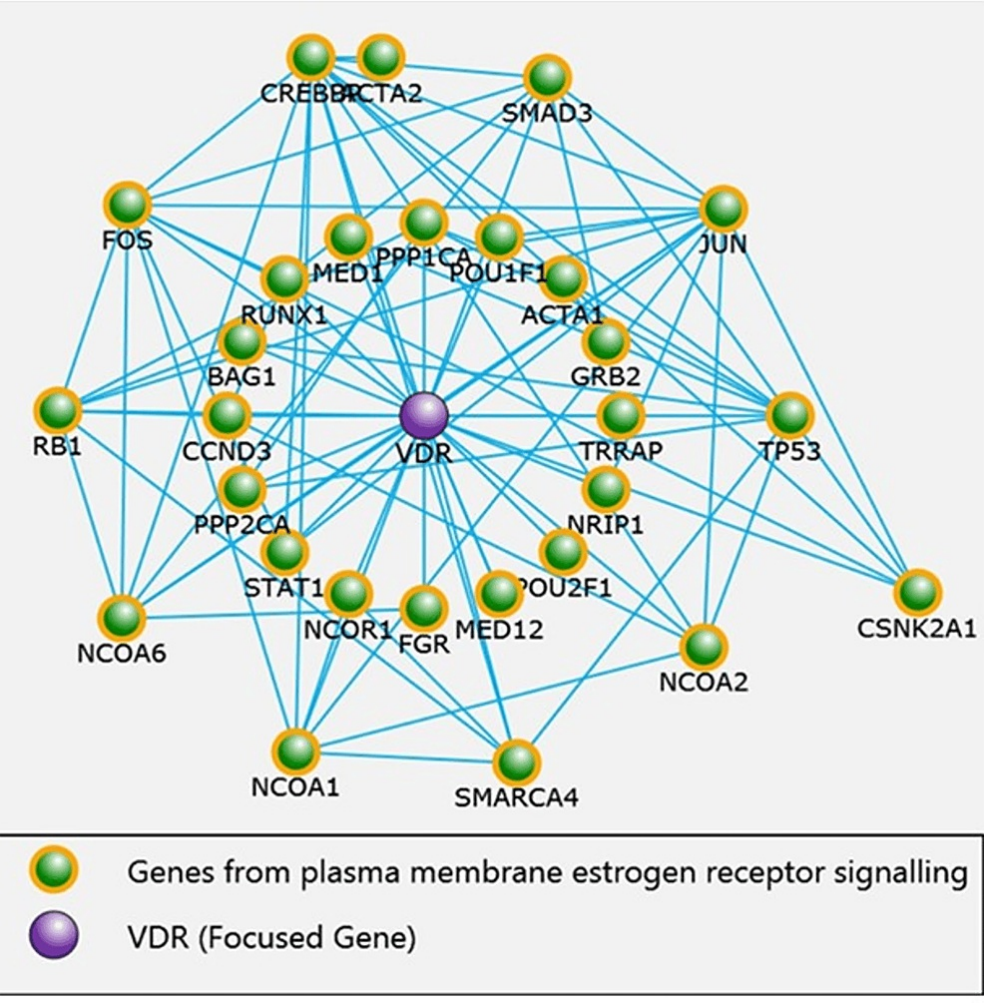
Genes enriched in plasma membrane estrogen receptor signalling pathway VDR (Vitamin D receptor)

The proteins enriched in plasma membrane estrogen receptor signalling were 29 in number with p < 0.001. These include VDR, SMAD3, NCOR1, CREBBP, NCOA1, STAT1, GRB2, PPP2CA, TP53, and NCOA2, which were enriched in plasma membrane estrogen receptor signalling pathway when VDR was made focused gene and directly interacting with more than twofold enrichment, as shown in Figure 3. It is worth mentioning that VDR, NCOR1, and SMAD3 were enriched in the ovarian infertility gene pathway.

## Discussion

This cross-sectional study revealed that a significantly low VDBP and VDR were in addition to low VD in UI females. We have found a mutation in exon 11 of the VDBP gene of infertile rs 4588 SNP, which may alter their protein function (Thr 436 Lys). The plasma membrane estrogen receptor signalling pathway was enriched in VD-deficient infertile females. VDR, SMAD3, NCOR1, CREBBP, NCOA1, STAT1, GRB2, PPP2CA, TP53, and NCOA2 were enriched in the plasma membrane estrogen receptor signalling pathway. It is worth mentioning that VDR, NCOR1, and SMAD3 were found to be enriched in the ovarian infertility genes pathway.

Our results are supported by a study [16], which indicated VDR gene polymorphisms as a contributing factor for infertility in patients with UI. Impaired VDR gene expression also affects the endometrial receptivity and implantation process through unknown underlying mechanisms. Our study exhibited VDR gene BsmI and TaqI polymorphisms as a substantial risk for UI, whereas the VDR gene Aa genotype in ApaI polymorphism is a protective factor. Another study mentioned the important effects of VD on endometrial receptivity and implantation; however, the detrimental effects on oocyte and embryo quality were observed due to its anti-oestrogenic effect [17]. Meanwhile, Jeremic et al. suggested measurements of VD in serum and follicular fluid as a complementary tool for routine assessment of embryos in UI patients undergoing IVF treatment [18].

VDR polymorphisms are associated with infertility and a decrease in folliculogenesis, oocyte yield, fertilization, and pregnancy rates after controlled ovarian stimulation (COS) responses in assisted reproductive techniques (ART) [19]. FokI is one of the most evaluated polymorphisms of the VDR gene. A polymorphic variant (FF) is generated from a change of T to C in the start codon sequence that is reduced by three amino acids and displays an amplified transcriptional deficit of the VDR protein in contrast to the long ff allele form. A study from India has established an association between the VDR gene (FokI, rs-2228570; C > T) polymorphisms and male factor infertility [20].

The identification of VDR polymorphisms specifically related to infertility and response to ovarian stimulation may help in a better understanding of processes for UI and affected ovarian reserves. Djurovic et al. [21] explored the association of VDR gene polymorphisms and haplotypes with UI. They examined the DNA of 117 patients with UI and compared it with 130 control fertile women. The results highlighted those changes in the expression, and the activity of the VDR gene affected the expression of VD-responsive genes, leading to altered immune effect and possible impact on reproduction. With two identified haplotypes, BAT was associated with an increased risk for the ability to conceive again, whereas haplotype BAT indicated a protective role for the ability to conceive for the first time (p < 0.05).

The presence of low VDBP in infertile females was indicated in a pilot study [22]. The observed association of rs 4588 SNP mutation in exon 11 of the VDBP gene of infertile subjects in our study corroborates with mortality due to COVID-19 with VDD and VDBP polymorphisms of rs7041 and rs4588 in the literature [23].

Given the growing concerns over the widespread and uncontrolled use of ART and intracytoplasmic sperm injection (ICSI), VD3 supplementation may turn out to be a simple and cheap clinical treatment for infertile couples.

Large-scale interaction networks encompass the results of experiments that help describe different biochemical interactions between genes and their encoding proteins [24].

The pathway analyses play an important role in appreciating biological steps involved in various disease processes. Hence, more compelling biomarkers can be identified using the dysregulated pathway [25].

We used a network-based method to determine the dysregulated pathways developed in VD-deficient infertile females, which may provide new discernments of the processes, leading to infertility in females. Estrogen facilitates its biological response through various potential cellular mechanisms, and this occurs mainly in two cellular ways, including receptors: genomic activity and rapid nongenomic effects [26].

It was described that the prompt response occurs within minutes in the process of therapy. Additionally, inhibition of the MAPK/ERK or AKT signalling pathway can block nongenomic effects. Initiating the commencement of these signalling pathway activities is closely related to GPR30-mediated plasma-membrane-associated processes [27].

In the current in silico interaction analysis, the plasma membrane estrogen receptor signalling pathway was found to be a dysregulated pathway based on the close interaction of VDR and VDBP genes. Our research identified several proteins enriched in estrogen receptor signalling pathways involving VDR, SMAD3, NCOR1, CREBBP, NCOA1, STAT1, GRB2, PPP2CA, TP53, and NCOA2.

Estrogen receptor protein is considered a main factor for estrogen action as it binds estrogens to originate tissue responses. These receptor proteins are of two types and include ERα and ERβ, and both have distinctive expression configurations [28].

It is worth mentioning, in this study, that VDR, NCOR1, and SMAD3 were also found to be enriched in ovarian infertility gene pathways. Fertility attributes in human inhabitants are controlled genetically [29].

Genomic-wide association studies (GWAS) identified 34 genome-wide significant signals for fertility in women with replication in deCODE data for the Icelandic population and in the Women’s Genome Health Study. The signals comprise association with intronic SNPs in the oestrogen receptor 1 (ESR1) gene that is also linked with the number of offspring [30].

Limitations

Studies suggest that BMI can modify response to VD supplementation leading to individuals with higher BMI. However, we did not study the effect association of BMI with metabolic disorders and alterations in VDBP, VDR, and VD. The study includes a relatively small sample size (40 VD-deficient fertile females and 40 UI female subjects). This might limit the generalizability of your findings to a broader population [19]. FokI is one of the most evaluated polymorphisms of the VDR gene. A polymorphic variant (FF) is generated from a change of T to C in the start codon sequence that is reduced by three amino acids. Our study has identified low levels of VDR and VDBP levels in UI females.

With this context, the identification of VDR, NCOR1, and SMAD3 in this study must be evaluated further to explore their role in therapies for VD-deficient females with infertility. VD screening and correction may add value to the outcome of patients receiving infertility treatments.

## Conclusions

The study revealed the potential implications of VDD and genetic variations in VDBP on reproductive mechanisms, highlighting the pathways and genes associated with unexplained infertility in females. This is supported by the identification of significantly reduced levels of VD, (VDBP) and VDR in UI females and a mutation in the VDBP gene at the rs 4588 SNP in exon 11 that affected the protein function (Thr 436 Lys). Furthermore, enrichment of the plasma membrane estrogen receptor signaling pathway in VD-deficient infertile females, especially VDR, SMAD3, NCOR1, CREBBP, NCOA1, STAT1, GRB2, PPP2CA, TP53, and NCOA2 directs towards a link of infertility with VDD.
